# Feeding and Processing Affect Meat Quality and Sensory Evaluation

**DOI:** 10.3390/foods14213756

**Published:** 2025-11-01

**Authors:** Sandra S. Q. Rodrigues

**Affiliations:** CIMO, LA SusTEC, Instituto Politécnico de Bragança, Campus de Santa Apolónia, 5300-253 Bragança, Portugal; srodrigues@ipb.pt

Paul D. Warriss [1] aptly described the evolution of agricultural systems as progressing through three distinct phases: first, meeting basic needs; second, improving production efficiency; and finally, enhancing product quality. Meat production follows this trajectory, and we are now in the phase where delivering high-quality products is paramount.

Meat quality [2] encompasses a set of desirable attributes that align with consumer expectations—appropriate conformation, nutritional value, sensory appeal, and safety. However, perceptions of meat quality vary widely depending on cultural background, personal experience, and the stakeholder’s role in the production chain—be it producer, retailer, or consumer. To meet these diverse expectations, meat production must strive for maximum output at minimal cost (efficiency), while simultaneously ensuring the highest standards of quality. However, this pursuit must never compromise animal welfare, health, or environmental sustainability.

Growth performance, carcass yield, and meat quality are influenced by a range of animal-related factors, breed and genetics [3,4,5], sex [6,7], age [8,9], and slaughter weight [10,11]. These elements must be carefully considered in management practices to optimize outcomes. External factors, including diet or feeding strategies [3,4,12,13,14], as well as processing techniques [15]—individually or in combination—can significantly affect meat quality and consumers’ sensory perception [16].

Feeding practices play a pivotal role in shaping various quality traits, from carcass composition and commercial value to nutritional content and organoleptic properties. Across species—from fish and monogastrics (including poultry) to ruminants—diverse strategies can be employed to produce meat that satisfies consumer expectations in terms of both nutrition and sensory appeal, while adhering to sustainable and animal-friendly principles. Approaches such as grass-fed systems, organic feed, and targeted supplementation are commonly used to modulate meat quality.

Since ancient times, humans have developed preservation techniques to extend the shelf life of food. In the absence of such methods, degradation due to microbial activity, chemical and enzymatic reactions, and physical changes become inevitable. Traditional preservation methods such as dehydration, smoking, brining, canning, fermentation, and refrigeration have increasingly been complemented or replaced by innovative techniques, including chemical and biological preservation and non-thermal technologies. Beyond extending shelf life, processing can also affect the technological and organoleptic characteristics of meat, influencing its flavor, texture, and overall consumer acceptance.

Meat quality and sensory appeal are central to consumer satisfaction, nutritional value, and innovation across the food industry. This Special Issue of *Foods*, entitled “Feeding and Processing Affect Meat Quality and Sensory Evaluation”, presents a diverse and insightful collection of studies that explore how feeding strategies and processing technologies influence the final attributes of meat products.

The published papers span a wide range of approaches—from dietary interventions using saline-grown oats and fruit powders to alternative protein sources like fly maggots and algae extracts. These studies underscore the intricate interplay between animal nutrition, biochemical composition, and post-harvest processing in determining meat texture, flavor, shelf life, and consumer acceptance. The findings presented offer practical insights for producers, researchers, and policymakers aiming to elevate meat quality through sustainable and science-driven methods. A summary of the published papers is presented below.

1. Natural Antioxidants as Nitrite Alternatives in Processed Meats

A major research front in this field is the search for natural alternatives to synthetic additives in processed meats. Two studies featured in this Special Issue advance the clean-label agenda by exploring plant-based antioxidants to replace synthetic nitrites in meat products.

Ferreira et al. investigated Bougainvillea spectabilis bracts, processed via three drying methods, as natural additives in cooked pork ham. Bougainvillea powder enhanced the antioxidant stability of the ham across all drying methods, as reflected by higher total polyphenol levels and improved results in DPPH, ABTS, and FRAP assays. At 0.1% inclusion, the powder significantly reduced lipid oxidation over eight weeks of storage, with no compromise in flavor, texture, or appearance. These findings position Bougainvillea spectabilis as a viable, consumer-friendly alternative to nitrites.

Manea et al. evaluated the use of blackcurrant, lingonberry, and sour cherry powders as natural antioxidants in nitrite-free salami formulations. Among them, blackcurrant powder showed the strongest oxidative protection, positioning it as a promising clean-label alternative to synthetic nitrites. The antioxidant efficacy varied by fruit type and was closely linked to phenolic content, with higher concentrations yielding better results. Additionally, smoked and cooked salami exhibited greater oxidative stability than scalded variants, underscoring the need to align antioxidant strategies with both ingredient functionality and processing conditions.

Together, these studies exemplify how targeted processing and phytochemical strategies can meet consumer demand for safer, additive-free meat products without sacrificing technological or sensory performance.

2. Sustainable Feeding and Meat Quality Enhancement

Sustainable feeding systems are increasingly recognized as key to reconciling animal performance with environmental stewardship. Two studies explore how alternative feed sources can improve meat quality while promoting environmental sustainability.

Xin et al. demonstrated that saline-grown oats enhance the nutritional profile of forage and improve meat quality in Qinghai Tibetan sheep. Using metabolomics, this study links elevated levels of protein, amino acids, and sugars in saline oats to improved meat texture and sensory quality, maintaining water-holding capacity. These findings suggest that saline-adapted crops can modulate muscle metabolism and offer a viable strategy for livestock production on marginal lands.

Leite et al. evaluated the inclusion of olive cake by-products in Bísaro pig diets. Incorporating centrifuged and pressed olive cake into dry-cured meat products did not alter their overall physicochemical properties, but notable differences emerged between product types in fat, protein, moisture, water activity, and haem pigments. Interactions between diet and product also affected parameters like aw, ash, and NaCl, indicating that products from the same muscle (LTL) can develop distinct profiles. Olive cake inclusion—especially at 25%—significantly increased n-3 fatty acid content and lowered the PUFA n-6/n-3 ratio, enhancing nutritional value without affecting other fatty acid fractions or quality indices. These distinctions may boost consumer appeal and support circular feed strategies.

3. Functional Feeds and Packaging Technologies

Two complementary studies highlight how feed innovation and smart packaging can synergistically improve meat quality and safety.

Fehri et al. assessed diets enriched with extruded linseed and *Padina pavonica* algae in meat rabbits. The functional feed improved the fatty acid profile of rabbit meat by reducing saturated fatty acids and the n-6/n-3 ratio, while increasing n-3 PUFA levels across two production cycles. Nutritional gains included higher levels of vitamin E, coenzyme Q10, and essential minerals, contributing to health-oriented meat products aligned with the One Health framework.

Castrica et al. investigated modified atmosphere packaging (MAP) with an active absorbent pad (aPAD) for omega-3-enriched rabbit meat. The aPAD reduced microbial growth and oxidative degradation over 21 days. Oxidative stability improved, especially in algae-fed groups, suggesting a synergistic effect of polyphenols and bioactive compounds in *Padina pavonica*, combined with the physical and chemical properties of aPAD. Sensory quality and consumer acceptability decreased over time, demonstrating the value of integrating functional feeds with smart packaging to extend shelf life, ensuring safety, and consumer acceptability.

4. Aquatic Protein Innovation

Liang et al. present a novel approach to crustacean nutrition by partially replacing conventional feed with housefly maggot larvae (HML) in adult Chinese mitten crabs. Over a 40-day trial, HML supplementation improved edible yield, antioxidant capacity, and sensory quality—particularly in female crabs. Nutritional enhancements included elevated essential amino acids, astaxanthin, and inosine monophosphate (IMP), contributing to better texture and flavor. These findings support insect-based feed strategies as sustainable, high-performance alternatives in aquaculture.

5. Phytogenic Additives in Poultry

Shu et al. investigated the effects of dietary curcumin (CUR) supplementation on chicken meat quality. At 150 mg/kg, CUR improved amino acid deposition, increased 5′-nucleotide levels, and enhanced the PUFA/SFA ratio by reducing saturated fats. Volatile compound analysis revealed elevated aldehyde concentrations, contributing to improved flavor. This study confirms curcumin’s potential as a natural additive to enhance both nutritional and sensory attributes in poultry meat.

**Closing Remarks**

As Guest Editor, I extend my sincere gratitude to all authors and reviewers and to the editorial team for their contributions to this Special Issue. The research presented here reflects a shared commitment to advancing meat science through sustainable feeding strategies and innovative processing techniques. It is my hope that this Issue will serve as both a reference and a catalyst for future innovations in food quality and sensory evaluation.

## Data Availability

Not applicable.
